# The 3rd annual American Society for Intercellular Communication (ASIC) meeting, 2023 conference report

**DOI:** 10.20517/evcna.2024.64

**Published:** 2024-12-05

**Authors:** Ashley E. Russell, Aurelio Lorico

**Affiliations:** ^1^Department of Biology, School of Science, Penn State Erie, The Behrend College, Erie, PA 16563, USA.; ^2^Department of Basic Sciences, College of Medicine, Touro University Nevada, Henderson, NV 89014, USA.

The third annual meeting for the **American Society of Intercellular Communication (ASIC)** was held in Potomac, Maryland, on October 12th-14th, 2023. The meeting brought together over 130 participants and was supported by nine sponsors, including Caerus, Ceres Nano, International Society for Extracellular Vesicles, Izon, Kinetic River, ONI, Particle Metrix, Serpin Pharma, and Virongy. The meeting was also supported by an NIH/NCATS R13 award. Fifty-two oral presentations were given over the course of the three-day meeting. Two poster sessions were held, one on the evening of the first day of the conference and the other on the evening of the second, with a total of thirty posters being presented. On the afternoon of the second day, a Grant Writing Workshop was held, which was specifically geared toward early career investigators and trainees.

Works presented at this meeting provided a comprehensive overview of the role of extracellular vesicles (EVs) as key modulators of intercellular communication for numerous disease contexts, including cancer and infectious, neurodegenerative, and cardiovascular diseases. Additionally, the potential utilization of EVs as both diagnostic and therapeutic tools was a main point of discussion throughout the meeting. Novel advancements in tools for EV visualization and analysis, including super-resolution imaging, were also described. All of the presentations summarized below appear in the order they were given at the meeting.

**Day 1 of Meeting:** The meeting began with opening remarks from the current president of ASIC, Fatah Kashanchi (George Mason University). The first session of the day was a NIH workship/Pre-Program session moderated by Uma Maheswari Deshetty (University of Nebraska) and Mariola Ferraro (University of Florida).

The first talk of the conference was given by Passley Hargrove-Grimes (NIH/NCATS) and focused on tissue chips/microphysiological systems for drug screening. Tissue chip technology is more physiologically relevant than conventional models, which is important since there are over 10,000 known diseases and only ~ 5% have treatments or cures, and 90% of therapeutic candidates that enter clinical trials fail. Tissue chips can even be used to inform clinical trial design and work toward the implementation of precision medicine by using pluripotent stem cells or primary tissues.

Christina Liu (NIH/NIGMS) provided an overview of different funding mechanisms that may interest the audience, including the R35, R21, NIH SuRE R16 program, and the RLI-S10 mechanism.

Martha Lundberg (NIH/NHLBI) spoke about the ways in which EV membranes and cargo could be altered, and the pros and cons of doing such work. Engineering EVs could change their stability, tropism, and release kinetics, which could affect their generation for commercial-scale manufacturing. This could have profound impacts on the use of EVs as therapeutics.

The last NIH Workshop speakers were Anu Puri (NIH/NCI) and Aniruddha Ganguly (NIH/NCI). Both spoke about training and funding opportunities through the NCI.

Uma Maheswari Deshetty (University of Nebraska) was the first pre-program speaker, and presented a study on ferroptosis, an intracellular iron-dependent form of cell death, implicated in HIV-associated neurocognitive disorder (HAND). HAND is still present in 30%-50% of HIV-1-infected individuals, despite effective suppression of viremia. They reported that HIV Tat induced both microglial activation and ferroptosis. Interestingly, microglia-derived EVs (MDEVs) stimulated by HIV-Tat contained ferroptosis mediators that could be taken up by neurons, causing neuronal injury. These *in vitro* findings were confirmed in HIV transgenic rats, with MDEVs isolated from the rat brains exhibiting packaging and release of ferroptosis mediators, accompanied by neuronal injury.

Navneet Dogra (Icahn School of Medicine) presented work on extracellular RNA analysis of EVs with particular emphasis on standardization and, especially, on the influence of short-read aligners on differential gene expression and gene set variation analysis and on different combinations of EV isolation methods. Enrichment of non-coding RNA (primarily lncRNA, tRNA, and Y RNA) in EVs was reported, with a negligible amount of miRNA (~ 2% of total). Different profiles were found across aligners, underscoring the impact of aligner choice on downstream analysis.

Suvendra Bhattacharyya (University of Nebraska) focused on the mechanism of selective packaging of miRNA into endosomes for subsequent extracellular export. They presented a model they developed, with endosomes isolated from macrophages, to follow miRNA packaging into endocytic organelles. By adding miRNA and the recombinant miRNA binding protein Human Antigen R (HuR) or miRNA and anti-HuR antibodies to isolated endosomes, they concluded that HuR drives miRNA import into endosomes.

Girish Neelakanta (University of Tennessee) reported on how the rickettsial pathogen *Anaplasma phagocytophilum* modulates intercellular communication in ticks. *Anaplasma* was found to induce the production of EVs in tick cells and enhance the expression of organic anion-transporting polypeptide (OATP). Interestingly, siRNA knockdown of OATP decreased pathogen survival in ticks and immunization of mice with OATP4 antibody prevented its transmission, suggesting the possibility of developing an anti-vector vaccine.

Mariola Ferraro (University of Florida) presented work on small EVs (sEVs) derived from salmonella-infected macrophages. Salmonella can be phagocytosed by macrophages and subsequently survive and proliferate. Infected macrophages then release sEVs that were shown to polarize macrophages and, when delivered intranasally in a mouse model, stimulate CD4^+^ T lymphocytes and induce the production of salmonella IgA and IgG. Further, splenocytes from these animals were found to have a strong TH1-biased recall response.

Chioma Okeoma (New York Medical College) focused on the role of extracellular condensates (ECs) in the persistence of HIV. Biomolecular condensates present in the extracellular milieu can form via liquid-liquid phase separation (LLPS) and may progress from dynamic, reversible aggregates to static, irreversible aggregates. ECs have been identified in several species from a variety of sources, including plasma, seminal plasma, prefrontal cortex, and the basal ganglia. ECs were found to activate latent HIV and induce changes to the transcriptome and secretome of infected cells, including microglia. These pathogenic ECs may also contribute to a weakening of the blood-brain barrier (BBB) by causing it to become leaky.

Theresa Whiteside (University of Pittsburgh School of Medicine) presented on sEVs found in the blood plasma of cancer patients. sEVs from cancer patients were found to induce apoptosis in activated T cells in a dose-dependent fashion. Interestingly, when using neutralizing antibodies for death receptors/ligands, apoptosis was only inhibited by approximately 10%. Caspase inhibition did not block T cell apoptosis after cancer-derived sEV exposure either; however, changes in survival proteins like BCL2, BCLXC, and BAX were observed. Tumor-derived EVs were found to enter T cells and alter mitochondrial integrity and function, as evidenced by the leakage of cytochrome C into the cytosol. Additionally, when T cells were co-incubated with melanoma-derived EVs, the unfolded protein response was found to be activated as well. These effects were predominately observed in CD8^+^ T cells, less so in CD4^+^ T cells, and very minimally in B or NK cells. Interestingly, EVs from the plasma of health donors or nonmalignant cells were found to be only minimally apoptotic to T cells.

Ryan Flynn (Harvard University) outlined the importance of glycosylation of RNAs on the cell surface. Glycosylation controls many processes, including how cells communicate with one another. The majority of work in this field has focused on glycosylation of proteins and lipids, but it was recently discovered that RNA, including small non-coding RNAs, could also be glycosylated. These glycoRNAs have been found on the surface of cells and can be complexed with RNA-binding proteins (RBPs). Interestingly, clusters of glycoRNA-RBPs have been observed on the surface of cells, and may play an important role in intercellular communication.

The last two speakers of the pre-program session provided overviews of new technologies available to EV researchers. Kevin Dolan (Particle Metrix) provided an overview of a new technical approach for using the ZetaView x30 instrument family to assess the colocalization of protein targets on EVs with fluorescence-based nanoparticle tracking analysis. Varun Sheel (ONI) discussed the design and use of a benchtop super-resolution microscope for EV characterization and visualization at the nanometer resolution and the availability of the CODI platform for data analysis.

The evening session on the first day, Biology of Intercellular Communication, was moderated by Aurelio Lorico (Touro University Nevada) and Theresa Whiteside (University of Pittsburgh Medical Center).

Aurelio Lorico provided opening comments to kick off the official start of the meeting, stressing the importance of ASIC as an interdisciplinary, collaborative scientific platform open to all. The presentation from the session’s keynote speaker, Susmita Sahoo (Ichan School of Medicine at Mount Sinai), was on blood EVs. EVs are typically released by cells circulating in the blood, and there is some evidence that EVs mediate some functions of the cells from which they are derived. It was found that CD34^+^ EVs enhance therapeutic recovery in a mouse model of hind limb infarction and improve cardiac function and fibrosis in mice. Interestingly, the loss of miR-126 results in the loss of the therapeutic potential of these CD34^+^ EVs. Further, circulating EVs from chronic kidney disease (CKD) patients were found to impair cardiomyocyte contractility.

Denis Corbeil (Technische Universitat Dresden) presented the results of studies on the role of membrane-enclosed structures in intercellular communication. CD133 has been identified as a marker of the plasma membrane in both neural and hematopoietic stem and progenitor cells. Interestingly, CD133 was found to be most highly concentrated in cellular protrusions, such as microvilli, primary cilium, and filipodium, and also associated with cholesterol/GM1-containing membrane microdomains and sEVs. CD133 was found to control microvillar structure and ciliary length, and increasing its cellular expression resulted in decreased EV release. Together, these data suggest that CD133 expression modulates cellular structure and communication.

Louise Laurent (University of California, San Diego) talked about the heterogeneity of EVs and their cargo. Immunomagnetic separation of EVs (CD63, CD9, and CD81) from conditioned cell culture media from colon cancer cells revealed that some EVs only express one canonical marker, but larger EVs are more likely to be double positive. This suggests that there exist different modes of EV biogenesis that produce EVs expressing varying combinations of these markers. Small RNA sequencing further revealed differences in cargo within these EV populations.

Jeffrey Franklin (Vanderbilt School of Medicine) gave the last talk of the day on large-scale production and purification of EVs and supermeres. Large-scale bioreactors were found to produce about 20-fold more EVs relative to traditional, 2D-grown DiFi colorectal cancer cells and these EVs were found to have similar secretome profiles. Fast protein liquid chromatography (FPLC) was also found to be a suitable alternative method for purifying supermeres from conditioned cell culture media, as opposed to differential ultracentrifugation. Interestingly, microglia exposed to supermeres obtained from cultured colorectal cells and colorectal cancer patients were found to elicit a cytokine response.

The evening concluded with a poster session, featuring the first half of the poster presentations.

**Day 2 of meeting:** The second day of the conference began with a session on Bacteria and EVs, moderated by Meta Kuehn (Duke University) and Ramin Hakami (George Mason University).

Meta Kuehn (Duke University) gave the first talk of the day on how bacteria release EVs with specific lipid profiles. Outer membrane vesicles (OMVs) derived from Gram-negative bacteria were found to modify bacterial outer membrane lipid composition after environmental shifts. Bacterial EVs (BEVs) may play important roles in bacterial adaptation in the environment and during infection.

Gagan Deep (Wake Forest School of Medicine) discussed the potential role of gut-derived BEVs in the gut microbiome-brain axis. Isolating BEVs from feces is a very complex technique due to the mixture of eukaryotic and bacterial vesicles present in the sample. Traditional density gradient ultracentrifugation is insufficient to achieve separation of these two populations; however, BEVs express lipopolysaccharide (LPS) while eukaryotic EVs express syntenin, so immunoprecipitation can be utilized to separate these two populations. Interestingly, dysbiotic BEVs were found to be enriched in L-histidinol. This functional metabolite is thought to be preferentially loaded into BEVs and was shown to impair long-term potentiation in hippocampal neurons, polarize microglia, and promote β-amyloid and tau aggregation. Therefore, these L-histidinol-containing BEVs may contribute to the pathogenesis of Alzheimer’s disease.

Marcela Rodriguez (Rutgers University School of Medicine) spoke about *Mycobacterium tuberculosis* (*Mtb*)-derived EVs and their role in pathogen-host communication. *Mtb* secretes hydrophobic siderophore mycobactin, suggesting that these EVs may contain iron. It was also found that these EVs can modulate immune cell function, indicating that there are further avenues of exploration to fully elucidate the role of EVs in *Mtb*-host interactions.

Ramin Hakami (George Mason University) provided new insights into the molecular mechanisms underlying the EV-mediated innate immune response to Gram-negative pathogenic bacteria. It was found that EVs from *Yersinia enterocolitica* induce the differentiation of naïve monocytes into macrophage-like cells, and these EVs also provide IL-6-dependent protection against infection in naïve cells.

Daniel Chiu (University of Washington) spoke about digital flow cytometry (DFC) as a tool to assess microparticle concentration, size, and heterogeneity. They described a newly developed flow platform, which uses a digital flow cytometer with 4 lasers and 12 detectors for the high-throughput multiplexed analysis of single EVs and particles. Their flow analyzer has single-molecule sensitivity. Dr. Chiu discussed potential applications ranging from EVs to RBPs, lipid nanoparticles, and viruses.

Heather Branscome (American Type Culture Collection) discussed large-scale manufacturing and characterization of EVs. The EV isolation protocol employed, based on tangential flow filtration, was found to be robust, reproducible, and had low lot-to-lot variability, yielding EVs that were functional *in vivo*. They also presented data suggesting that hTERT mesenchymal stem cell EVs may promote the repair of damaged retinal cells.

Kevin Weyant (Versatope Therapeutics) presented data on vaccines based on bacterial OMVs. Their rationale is that recombinant OMVs displaying antigens are potent adjuvant formulations to elicit an immune response against bacterial and viral pathogens. Data were presented for recombinant OMVs displaying an influenza A protein domain, suggesting that it might be a universal flu vaccine that prevents influenza pandemics while avoiding yearly vaccination. To expand the versatility of OMV-based vaccines, they have also developed an avidin-based vaccine antigen cross-linking (AvidVax) platform, whereby any biotinylated antigen can be attached to OMVs for improved immune potency.

Gerardo Mauleon (Izon Science) discussed a customized workflow for EV diagnostics and therapeutics, scaling EV isolation through a platform named qEVPurepath, based on GEN 2 qEV columns and qEV magnetic concentration kit. They propose that the EV field can benefit from the customizable qEVPurepath for therapeutics by bridging the current gap and challenges related to EV isolation.

The second morning session focused on EVs and the central nervous system (CNS) and was moderated by Ashley Russell (Penn State Erie, The Behrend College) and Navneet Dhillon (University of Kansas Medical Center).

The first presentation of the session was given by Partha Chandra (Tulane University), who discussed the use of plasma EVs as biomarkers for neuronal dysfunction in simian immunodeficiency virus (SIV)-infected rhesus macaques. SIV infection may weaken the BBB and impair mitochondrial function in brain microvessels. If the BBB is weakened, CNS-derived EVs may be more readily available in the circulation. Indeed, plasma EV protein profiles were able to differentiate between infected vs. uninfected macaques, and neuropathology-associated proteins were high in EVs from the infected animals. Further, HIV^+^ EVs were found to be taken up by mouse brain microvessels *ex vivo* and resulted in mitochondrial-dependent endothelial dysfunction.

Juliet Santiago (Emory University) shared recent work on MDEVs. Microglia can become disease-associated microglia and take on pro- or anti-inflammatory properties. Exposure to various inflammatory stimuli caused microglia to release EVs with pro-inflammatory mediators, microRNAs, and pathogenic proteins. Further, exposing microglia to compounds that induce homeostatic, pro-, or anti-inflammatory phenotypes leads to the release of EVs with state-specific proteomic and transcriptomic profiles.

Ursula Sandau (Oregon Health and Science University) presented the use of extracellular RNAs (exRNAs) as biomarkers for Alzheimer’s disease. Cerebrospinal fluid (CSF) was fractionated using size exclusion chromatography and found to contain both EVs, which are enriched for exosome and microvesicle markers, and smaller nanoparticles. Multiplex bead-based flow cytometry revealed distinct markers for vesicles from patients with Alzheimer’s versus Parkinson’s disease, both of which were differentiated from age-matched controls.

Seung-Wan Yoo (Johns Hopkins University) shared data showing that neutral sphingomyelinase 2 (nSMase2), found on the exterior surface of lysosomal/endosomal compartments and the inner leaflet of the plasma membrane, was identified as a critical component of HIV assembly and maturation. Many compounds can activate nSMase2 and HIV infection was found to increase nSMase2 in H9 cells. Inhibition of nSMase2 reduced plasma HIV-1 levels, reduced or prevented viral rebound, and selectively killed HIV-1-infected cells, indicating that it may be an important potential target for HIV treatment.

The last presentation of this session was delivered by Susmita Sil (University of Nebraska Medical Center) and also focused on HIV-1. As people living with HIV continue to age, they are more at risk for developing neurocognitive disorders. EVs derived from astrocytes exposed to HIV-1 Tat were shown to contain β-amyloid in their lumen. Interestingly, these EVs were shown to induce dendritic injury in neurons and Alzheimer’s-like pathology.

The first afternoon session of the second day of the conference, entitled “EVs and Cancer”, was moderated by Lucia Languino (Thomas Jefferson University) and Jonathan Geiger (University of North Dakota).

Lucia Languino discussed the role of the alphaV-beta3 integrin (αVβ3)/Nogo receptor 2 (NgR2) complex as a key regulator of pro-tumorigenic cargo loading of EVs. The GPI-linked surface molecule NgR2 is upregulated by αVβ3, to which it associates. They found that αVβ3^+^ EVs released by prostate cancer cells have high levels of NgR2 and programmed death-ligand 1 (PD-L1), a pro-tumorigenic molecule and a key player in immune evasion, respectively. Intra-tumoral injection of Nrg2^+^ EVs promoted tumor growth and induced differentiation. To confirm the clinical relevance of the findings, Dr. Languino showed that EVs isolated from the blood plasma of metastatic prostate cancer patients were enriched in both αVβ3 and NgR2.

Lance Liotta (George Mason University) reported an investigation on the potential role of EVs isolated from tumor interstitial fluid (TIF) as cancer biomarkers. TIF EVs are released from many cell types present in the tumor microenvironment and the cell source of EVs can be distinguished by their molecular proteomic analysis. Interestingly, anti-PDL1 immunotherapy of tumors increased the abundance of TIF EVs derived from immune and stromal cells. They concluded that TIF EVs constitute a new way to probe the *in vivo* state of tumor immunotherapy without the need for surgical tumor biopsy.

Susanne Gabrielsson (Karolinska Institute) shared recent data on the role of programmed cell death protein 1 (PD-1) and PD-L1 on EVs in immune therapy. EVs from antigen-presenting cells have a potential cancer immunotherapeutic role because they stimulate tumor-specific activity in mice. They showed that EVs from bone marrow-derived dendritic cells can stimulate B cells, T cells, and natural killer (NK) cells, resulting in increased immune cell infiltration in tumors and decreased tumor growth. They also reported that PD-L1-knockout EVs induce a higher IgG2 response early and a stronger CD8^+^ T cell response in the spleen and more T cell infiltration in tumors.

My Mahoney (Thomas Jefferson University) discussed the role of the cadherin and cancer biomarker desmoglein 2 (Dsg2) in head and neck squamous cell carcinoma (HNSCC), especially in the context of therapy with immune checkpoint inhibitors (ICI). In human papillomavirus-negative HNSCCs, Dsg2 expression correlated with the pathological stage and lymphovascular invasion, and high levels of Dsg2 transcripts were observed at the leading edge of aggressive tumors. Along the same line, anti-Dsg2 antibodies abrogated tumor xenograft growth and tumors of patients treated with ICI had lower Dsg2 levels.

Muller Fabbri (Children’s National Hospital) focused their presentation on the role of NK-derived EVs in the immunotherapy of neuroblastoma. The overarching goal was to develop NK EVs as a new anti-cancer therapy. Compared with therapies with NK cells, NK EVs have the advantage of minor side effects (e.g., cytokine storm), the possibility to reach pharmacological sanctuaries, and reduced immune-escape risk.

The second afternoon session was focused on EVs and Therapeutics and was moderated by Mark Santos (Touro University Nevada) and Chioma Okeoma (New York Medical College).

The keynote speaker for this session was Thomas Tuschl (Rockefeller University), who presented work on sequencing-based extracellular RNA profiling during pregnancy. A small pilot study revealed that unsupervised clustering could separate serum and plasma miRNA profiles. Interestingly, during pregnancy, there are different miRNA profiles relative to non-pregnant female controls. This is likely due, in part, to specific families of miRNAs being upregulated during pregnancy, especially those from the placenta. It is important to note that the way in which RNA sequencing data are analyzed, low abundance sequences can sometimes be lost, so careful attention must be given to how this is executed.

Jonathan Geiger (University of North Dakota) focused on weak base drugs and the lysosomal stress response. Lysosomal iron and the greater lysosomal system can be greatly impacted by the acidity of drugs, including weak base drugs such as chloroquine and hydroxychloroquine. Although different weak base drugs can have a variety of mechanisms of action, they can result in ion trapping, which increases the pH of lysosomes and kicks iron out into the cytosol and mitochondria, which can lead to increased levels of cytosolic and mitochondrial reactive oxygen species, resulting in mitochondrial membrane depolarization and cellular death.

Piul Rabbani (New York University) presented work on the use of EV-encapsulated hydrogel topical treatments for diabetic wound closure. Diabetic wounds are chronic and do not close easily on their own and present large economic and health burdens worldwide. Mesenchymal stem cell-derived EVs were found to promote diabetic wound healing; however, controlling the dose delivered to the wounds was challenging. Through collaborations with engineers, a hydrogel was created that forms upon cooling soluble nanofibers to skin surface temperature. Upon loading the EVs into the hydrogel, they became interspersed throughout the fibers. This hydrogel was then applied onto the mouse skin, where it helped wound repair with few side effects. It was found that EVs were present in the wound for several days and no off-target delivery of EVs was observed.

Dirk Dittmer (University of North Carolina, Chapel Hill) gave the last presentation of the session, discussing EV structure. EVs are small and round, with proteins protruding from their surface. Super-resolution microscopy (dSTORM) enables the visualization and characterization of EVs in 2D, with recent efforts now facilitating their 3D visualization. Membrane dye is only observed in the EV membrane and typically makes EVs look donut-shaped in 3D. Interestingly, it was found that EV membranes are not homogeneous, but are characterized by subdomains with distinct membrane and protein compositions.

The second day of the meeting concluded with a second poster session, featuring the other half of the poster presentations.

**Day 3 of meeting:** The first morning session, EVs and Infectious Disease, was moderated by Uta Erdbruegger (University of Virginia) and Michael Graner (University of Colorado).

Andrew Mouland (McGill University) talked about virus-mediated assembly of membrane-less biomolecular condensates of proteins and nucleic acids. They reported that pan-virus RNA-binding protein motifs are juxtaposed by disordered domains and pan-retroviral nucleocapsid proteins undergo zinc-dependent LLPS. Additionally, HIV-1 nucleocapsid-induced LLPS mediates viral RNA positioning and trafficking and HIV-1 nucleocapsid is the central scaffolding condensate forming HIV-1 biomolecular condensates.

Michael Graner (University of Colorado) reported on the production of EVs from a recurrent malignant chordoma, a primary sarcoma of the spine and skull base. Malignant chordoma-derived EVs may play an important role in the remodeling of the extracellular matrix in the tumor microenvironment. Specifically, they described that chordoma EVs express five different integrin subunits and promote tumor cell migration and proteomic changes in osteoblasts, possibly being involved in tumor invasion. Interestingly, blocking TGF-β on EVs reduced cellular ATP levels.

Xuesong Chen (University of North Dakota) reported on the role of SLC38A9, an endolysosome-resident arginine sensor in SARS-CoV2 entry. SARS-CoV2 viral entry depends on the cleavage of Spike (S) protein into S1 and S2 by cellular proteases of the host. They reported that S1 enters into endolysosomes and induces their deacidification and that SLC38A9 mediates S1-induced endolysosome deacidification. In fact, SLC38A9 knockdown prevents S1-induced endolysosome deacidification and attenuates SARS-CoV2 viral entry. Thus, S1 interaction with SLC38A9 could lead to the escape of SARS-CoV2 from degradation by endolysosomes.

Rakesh Singh (Touro University Nevada) studied a cohort of SARS-CoV2-infected subjects for up to 24 weeks, using dSTORM to analyze two key viral proteins, spike and nucleocapsid, in EVs isolated from blood plasma. They developed a new method, based on EV permeabilization, to image both surface and cargo proteins. Surface and intra-vesicular spike and intra-vesicular nucleocapsid were detected in all infected subjects, with a considerable decrease in both antigens between 8 and 12 weeks post-infection. The decrease in spike levels appeared to be age-dependent, suggesting a faster waning of immunity with age and, consequently, the need for repeated boosting in older subjects.

Navneet Dhillon (University of Kansas Medical Center) gave the last presentation of the first morning session and presented data on blood plasma EVs and COVID-19-associated cardiopulmonary dysfunction. They reported that pro-inflammatory and coagulation markers are higher in EVs from moderately and critically ill COVID-19 patients and that in the general circulation, soluble or EV-linked Spike protein persists in individuals with long COVID. Moreover, exposure of Sprague-Dawley rats to circulating EVs from acutely infected COVID-19 patients increased pulmonary microvascular injury and cardiovascular complications.

The last session of the conference was moderated by Xuesong Chen (University of North Dakota School of Medicine and Health Sciences) and Navneet Dogra (Icahn School of Medicine at Mount Sinai).

Gerardo Kaplan (US Food and Drug Administration) spoke about the potential role of EVs in mediating hepatitis A infection. It was found that hepatitis A-infected cells produce both viral particles and EVs, and the vesicles express the canonical EV markers, CD63 and CD81, whereas the viral particles do not. Further, the lipid receptors hepatitis A virus cellular receptor 1 (HAVCR1) and NPC intracellular cholesterol transporter 1 (NPC1) were found to be required for endosomal fusion and cargo delivery, and HAVCR1 was shown to serve as a receptor for hepatitis A EVs and mediate infection.

Hameeda Sultana (University of Tennessee) focused on a novel tick EV cement protein and its potential as a vaccine candidate to block vector-borne diseases. EVs in tick saliva, introduced at the feeding site on a host, were found to modulate cytokine signaling, as well as wound healing and repair in human keratinocytes. Cement proteins, secreted by ticks during feeding, play a crucial role in anchoring tick mouthparts to the host’s skin. Notably, silencing the expression of this cement protein in tick salivary EVs reduced the viral load of the Langat virus (LGTV), a tick-borne virus, in host mice.

Sabita Roy (University of Miami Health System) presented on morphine and its effects on EV-mediated immune modulation in intestinal organoids. There is a strong interplay between the gut microbiome, intestinal epithelium, and the immune system, and EVs have been shown to regulate immune responses and cellular function. It was found that EVs derived from both intestinal and colonic organoids modulated the inflammatory cytokine response in recipient cells and were able to alleviate symptoms in a mouse model of inflammatory bowel disease. Interestingly, morphine treatment abolished these effects.

Priyanka Gokulnath (Harvard University) spoke about the role of EVs in heart failure. In the United States, 6.5 million people suffer from one of two types of heart failure: heart failure with reduced ejection fraction (HFrEF) or heart failure with preserved ejection fraction (HFpEF). Arrhythmia is commonly observed in HFrEF patients and approximately 50% of heart failure mortality is due to arrhythmia. Induced pluripotent stem-cell-derived cardiomyocytes (iPSC-CMs) treated with EVs isolated from HFrEF patients showed a slightly altered duration of action potential relative to cells treated with EVs from healthy controls. Further, small RNA sequencing of the donor EVs revealed different miRNA profiles between HFrEF and controls, as did transcriptomic profiling of the recipient iPSC-CMs. These analyses revealed several miRNA and mRNA targets that converge on pathways that promote arrhythmia.

Uta Erdbrueger (University of Virginia) focused on the variability and temporal patterns of urinary EVs (uEVs) in healthy subjects. uEVs have the potential to be easily available diagnostic and prognostic biomarkers and most are produced by the kidney, bladder, and the cells in the urine itself, including leukocytes and bacteria. uEVs have also been shown to mirror changes in the kidney and there is a good correlation between uEV excretion rate and nephron mass. Interestingly, the kidney has its own circadian clock and a strong understanding of the variability of uEVs derived from healthy patients is necessary to understand and implement their use as biomarkers for various diseases. Concentrations of uEVs and their cargo were found to vary greatly, but not significantly, over the course of a 24 h period.

The last talk of the meeting was given by Leonid Margolis (NIH/MICHD). This talk focused on how much the intercellular communication field still needs to learn about EVs, as they are extremely complex and can be very challenging to study. For example, even a single cell can release a very heterogeneous population of EVs.

The meeting concluded with closing remarks from several members of the ASIC organizing committee and awards were presented for the top three oral and poster presentations. The top three oral presentations were Piul Rabbani (New York University), Chioma Okeoma (New York Medical Center), and Juliet Santiago (Emory University). The top three poster presentations were Emily Stack (NIH), Purva Gade (George Mason University), and Kaitlynn Slattery (NIH).

ASIC President Fatah Kashanchi (George Mason University) thanked the Organizing Committee for their hard work in bringing this meeting together, especially for preserving the hybrid approach that allowed for richer scientific interaction, and the constant support of the other ASIC founders Leonid Margolis (National Institute of Child Health and Human Development, NIH), Julie Saugstad (Oregon Health & Science University), Meta Kuehn (Duke University), Michael Graner (University of Colorado Anschutz), and Janusz Rak (McGill University) with a special note of appreciation to Gwen Cox for her consistent dedication in ensuring the event ran smoothly and efficiently*.*

## DECLARATIONS

### Copyright

© The Author(s) 2024.

